# Age‐related changes in gut microbiota and their correlation with psychosocial factors in community‐dwelling older adults

**DOI:** 10.1002/alz70857_106164

**Published:** 2025-12-26

**Authors:** Nurul Izzati Ahmad Fadzuli, Siong Meng Lim, Abu Bakar Abdul Majeed, Maw Pin Tan, Khor Min Hui, Ai Huey Tan, Chun Wie Chong, Kalavathy Ramasamy

**Affiliations:** ^1^ Universiti Teknologi MARA (UiTM), Puncak Alam, Selangor, Malaysia; ^2^ Brain Degeneration and Therapeutics Group, Faculty of Pharmacy, Universiti Teknologi MARA (UiTM), Puncak Alam, Selangor, Malaysia; ^3^ University of Malaya, Kuala Lumpur, Malaysia; ^4^ Monash University Malaysia, Bandar Sunway, Selangor, Malaysia; ^5^ Collaborative Drug Discovery Research (CDDR) Group, Faculty of Pharmacy, Universiti Teknologi MARA (UiTM), Puncak Alam, Selangor, Malaysia

## Abstract

**Background:**

The process of ageing not only leads to physiological but also psychological changes. Psychosocial factors such as loneliness, anxiety and depression, which can have profound effects on one's health, are particularly prevalent among older adults. Whilst it is known that ageing is associated with a shift in microbial balance, recent evidence suggests that these psychological factors may also influence gut microbiota composition. An imbalanced gut microbiota or dysbiosis, has been linked to various age‐related cognitive decline. As such, this study was undertaken to uncover the interplay between psychosocial factors and gut microbiota in older adults.

**Method:**

To this end, community‐dwelling individuals aged ≥65 were recruited. A total of 127 stool samples were collected, homogenised, and DNA was extracted. PCR amplification was performed before preparing the DNA library. Next, 16S rRNA gene sequencing was conducted on the V3‐V4 hypervariable regions using the Novaseq6000 Illumina platform. Statistical analyses were performed using GraphPad Prism 9.0 and R software.

**Result:**

The Actinobacteriota and Firmicutes phyla, which consist of potentially beneficial bacteria, were significantly higher in the 65‐74 age group, whereas the Proteobacteria phylum, a signature of dysbiosis, was significantly higher in the ≥75 age group. A similar pattern was also observed at the genus level whereby the 65‐74 age group was presented with higher *Acidaminococcus*, *Ligilactobacillus*, *Megamonas*, *Megasphaera*, *Collinsella*, *Agathobacter*, *Bifidobacterium*, *Dorea*, and *Faecalibacterium* genera, whereas the ≥75 age group exhibited higher *Escherichia‐Shigella*, *Intestinibacter*, and *Clostridium sensu stricto 1* (i.e., opportunistic pathogens). Notably, the *Megasphaera* genus was found to be negatively correlated with loneliness (*r* = ‐0.2904, *p* = 0.0009) and depression (*r* = ‐0.2369, *p* = 0.0073). The *Faecalibacterium* genus was also negatively correlated with anxiety (*r* = ‐0.2388, *p* = 0.0068) and loneliness (*r* = ‐0.2341, *p* = 0.0081) whilst the *Ligilactobacillus* genus showed a negative correlation with loneliness (*r* = ‐0.2099, *p* = 0.0178).

**Conclusion:**

This study unveiled how ageing may disrupt gut microbiota balance, with physiological and psychological factors further influencing its composition. Future research should assess their impact on health and see if restoration of gut microbiota is useful.

